# Think Beyond Particle Cytotoxicity: When Self-Cellular Components Released After Immunogenic Cell Death Explain Chronic Disease Development

**DOI:** 10.3389/ftox.2022.887228

**Published:** 2022-07-01

**Authors:** Riccardo Leinardi, Chiara Longo Sanchez-Calero, François Huaux

**Affiliations:** Louvain Centre for Toxicology and Applied Pharmacology (LTAP), Institut de Recherche Experimentale et Clinique (IREC), Université Catholique de Louvain (UCLouvain), Bruxelles, Belgium

**Keywords:** silica, immunogenic cell death, DAMP, DNA, inflammation, cancer, autoimmune diseases

## Abstract

The prolonged perturbation of the immune system following the release of a plethora of self-molecules (known as damage-associated molecular patterns, DAMPs) by stressed or dying cells triggers acute and chronic pathological responses. DAMPs are commonly released after plasma membrane damage or complete rupture due to immunogenic cell death (ICD), upon numerous stressors including infectious and toxic agents. The set of DAMPs released after ICD include mature proinflammatory cytokines and alarmins, but also polymeric macromolecules. These self-intracellular components are recognized by injured and healthy surrounding cells *via* innate receptors, and induce upregulation of stress-response mechanisms, including inflammation. In this review, by overstepping the simple toxicological evaluation, we apply ICD and DAMP concepts to silica cytotoxicity, providing new insights on the mechanisms driving the progress and/or the exacerbation of certain SiO_2_–related pathologies. Finally, by proposing self-DNA as new crucial DAMP, we aim to pave the way for the development of innovative and easy-to-perform predictive tests to better identify the hazard of fine and ultrafine silica particles. Importantly, such mechanisms could be extended to nano/micro plastics and diesel particles, providing strategic advice and reports on their health issues.

## Introduction

Cell death is a process that can occur in multiple modalities in response to different stimuli. During the past few decades, guidelines concerning the definition and interpretation of cell death from morphological, biochemical and functional points of view were formulated and continuously updated by the constant discovery of new cell death pathways and molecular mechanisms (Galluzzi et al., 2018).

The process of regulated cell death (RCD) is a controlled chain of biochemical events finally leading to cell death, orchestrated by a complex signaling cascade and involving specific molecular effectors. RCD is crucial in the life of multicellular organisms, since it allows preserving tissue homeostasis and ensures several physiological processes to take place. In addition, it can be related to inflammation, immunological diseases, and cancer development (Hu et al., 2021). For at least two decades, apoptosis was considered the only form of RCD in opposition to necrosis, a supposedly accidental and non-regulated cell death mechanism following physicochemical insults. Because of the rapid recognition and clearance of apoptotic cells, apoptosis is classically recognized to be not inflammatory in most settings. This oversimplified view has been deeply revised based on new molecular pathways. It is now recognized that apoptosis, in certain conditions, causes specific inflammatory responses (Obeid et al., 2007) and plays important roles in the modulation of the adaptive immune system (MacDonald et al., 1988; Teh et al., 1988; Nemazee and Burki 1989; Hartley et al., 1991). Additionally, researchers clearly identified different modalities of highly regulated necrosis, including necroptosis and pyroptosis. These modalities commonly induce inflammation by promoting the release of a plethora of proinflammatory mediators and self-intracellular components known as damage-associated molecular patterns (DAMPs). These molecules manage the activation of innate (monocytes, macrophages and dendritic cells) and adaptive (T and B lymphocytes) immune cells by interacting with pattern recognition receptors (or PRRs as TREM and GPCR receptors) and non-PRR transmembrane proteins (Gong et al., 2020). The engagement of PRRs by DAMPs triggers the production of master proinflammatory cytokines such as IL-1 and TNF, which orchestrate immune system activation (Roh and Sohn 2018).

It is important to note that this accepted concept was already proposed almost 30 years ago by Polly Matzinger in her “danger theory”, suggesting that immune responses are induced by “danger” or “alarm” self-signals (Matzinger 1994; Matzinger 2002). Contrary to the classical “self-/non-self” discrimination theory of immune responses that dominated immunology for over 60 years, the danger model holds that immune responses depend on the release of specific danger signals from damaged cells. Inspired by this hypothesis, the notion of immunogenic cell death (ICD) was defined by the Nomenclature Committee on Cell Death as a subtype of regulated cell death that alerts the innate and adaptive immune system in “immunocompetent syngeneic hosts” (Casares et al., 2005; Galluzzi et al., 2020). Historically, the discovery of inflammasome complexes boosted our comprehension of the key role of ICD during immune responses (Schroder and Tschopp 2010). The identified triggers inducing inflammasomes activation and mature proinflammatory cytokine release (IL-1β/IL-18) were extremely diverse, and included pathogen-derived ligands (such as microbial cell wall components and nonself-nucleic acids), environmental crystalline pollutants (e.g., silica, asbestos, and alum) and endogenous molecules (ATP, serum amyloid A, and uric acid crystals). In virtue of this, ICD is now identified as the result of 1) defense mechanisms against pathogens (pathogen-driven ICD) and/or (ii) cytotoxicity elicited by conventional chemotherapeutics, anticancer agents, and physical damage including ionizing radiations, photodynamic therapies and heat-shock treatments (Fucikova et al., 2020). These stressors can trigger different ICD modalities, each one connected with the release of a specific panel of damage signals (Galluzzi et al., 2017). The occurrence of a specific modality rather than another relies on the stimulus (or stimuli) inducing cells to die and, in addition, on the possible crosstalk/overlap between distinct intracellular pathways activated by simultaneous stimuli (Roh and Sohn 2018). Besides passive secretion following plasma membrane destabilization/lysis during ICD, DAMPs are actively released from living cells via different mechanisms, including lysosomal exocytosis and exosomes. Some of these mechanisms are shared among different DAMPs, while others, such as ATP channels, are DAMP-specific (Murao et al., 2021).

Alarmins, as advanced by Joost Oppenheim in 2005, are a class of immune activating constitutively-expressed proteins rapidly released in response to infection, cell injury or necrosis (Oppenheim and Yang 2005; Yang D. et al., 2017). These very early proinflammatory DAMPs (mainly comprising HMGB1, IL-1α and IL-33) recruit and activate leukocytes and initiate both innate and adaptive immune responses (Bianchi 2007; Yang et al., 2013; Bertheloot and Latz 2017). Currently, one of the most challenged and studied damage signals is double-strand self-DNA (self-DNA). Likewise alarmins, self-DNA is considered as a surrogate biomarker of ICD (Galluzzi et al., 2020). In this context, recent experimental data evidenced that nuclear and mitochondrial self-DNA fragments released in the cytosol after cell damage or endocytosed from the extracellular environment (extracellular self-DNA) are sensed by cytoplasmic DNA sensors. These DNA-binding proteins play crucial roles in the activation of several responses driving the production of key inflammatory cytokines such as IFN-I, finally leading to ICD, inflammation and cancer (Rathinam et al., 2010; Li et al., 2013; Briard et al., 2020; Gong et al., 2020).

ICD process is induced by several types of inorganic fine and ultrafine particles, including silica. Many studies suggest a clear association between crystalline silica exposure and increased incidence of several local and systemic diseases including silicosis, obstructive lung diseases, lung cancer and autoimmune diseases (IARC 2012). Interestingly, early responses following the prolonged silica inhalation include cytotoxicity and immune and non-immune cells activation (Hubbard et al., 2002; Porter et al., 2004). Because cytotoxicity can result in cell death and considering that certain cell death modalities can modulate the activation of the immune system, we propose in this review that ICD and ICD-associated DAMPs trigger, sustain, and amplify the detrimental effect of silica. ICD, in our view, represents the molecular link between cell death modalities and the development of silica-induced pathologies.

Here, we detail the current understanding of the main ICD subtypes induced by amorphous and crystalline silica and propose a new link between particle-induced cell death, immune response modulation, and development of silica-associated chronic inflammation, fibrosis, cancer and autoimmunity. Overall, the aim of this review is to advance the knowledge on the mechanisms triggering the progress of localized and systemic pathologies induced by the prolonged exposure to silica, providing an updated mechanistic model based on self-DNA signaling expanding the current schema for silica-related pathogenicity.

## The “Previous” Model Elucidating the Proinflammatory Activity of Silica

Occupational exposure to crystalline silica dust has been identified as a major cause of chronic inflammation, fibrosis, cancer and autoimmune diseases (Hoy and Chambers 2020; Togbe et al., 2020). Compared with crystalline, amorphous silica has been generally considered safe. Indeed, the fact that amorphous silicas are more soluble and less biopersistent supports the hypothesis that the pathogenic potential associated to amorphous silica is way less alarming with respect to crystalline particles (Johnston et al., 2000; Arts et al., 2007). However, it was reported that amorphous SiO_2_ nanoparticles (primary size ca. 100 nm) could trigger *in vivo* acute inflammatory responses (Kusaka et al., 2014; Nemmar et al., 2016; Wang et al., 2020). In addition, recent investigations suggested that crystallinity *per se* does not play a significant role in the pathogenicity of silica, which may be rather related to specific surface moieties (namely “nearly free silanols”, NFS) detectable in both amorphous and crystalline silicas (Turci et al., 2016; Pavan et al., 2020). Because of this, the current paradigm of silica toxicity should be carefully re-evaluated.

Until now, it has been argued that inhaled silica particles reach the bronchioles and the pulmonary alveoli, where they are engulfed by alveolar macrophages (AM). Particle detection is thought to take place via scavenger receptor (SR) sensing, a subfamily of PRRs expressed by professional innate immune cells (Poinen-Rughooputh et al., 2016; Pollard 2016; Hoy and Chambers 2020). Indeed, it was observed that the binding of crystalline silica to SR induced the release of proinflammatory mediators by activated cells, proposing a key role for SR in the activation of intracellular signaling following binding of environmental particles (Thakur et al., 2008). Among SR, several studies support SR-A1, SR-A6 (also known as MARCO) and SR-B1 as crucial in silica recognition, and macrophage activation (Hamilton et al., 2006; Thakur et al., 2009b; Nishijima et al., 2017; Tsugita et al., 2017). The interaction between particles and SR-A/SR-B in macrophages activates NF-κB and AP-1 transcription factors and MAPK and MerTK intracellular transduction pathways (Nishijima et al., 2017). These processes are directly associated with the rapid production and secretion of proinflammatory cytokines and chemokines (TNF-α, CXCL1, CXCL10 and CCL2) and ROS generation (Morgan and Liu 2011; Zhang et al., 2016; Liu et al., 2017). However, contradicting observations from SR-A1 and SR-A6-deficient macrophages and murine models evidenced that the proinflammatory response to silica is exacerbated in the absence of these receptors. This suggests that particle sensing and internalization can occur via receptor-independent pathways and, furthermore, that the receptors-driven sensing might rather reduce the proinflammatory outcome (Arredouani et al., 2004; Beamer and Holian 2005; Thakur et al., 2009a). In addition to macrophages, crystalline silica stimulates epithelial cells to produce and release cytokines (i.e. IL-8, IL-25 and TSLP) *via* unclear mechanisms, likely involving the activation of SFK and ERK1/2 pathways and not requiring particle uptake (Ovrevik et al., 2006; Unno et al., 2020). Furthermore, in physiological conditions, AM are maintained in a quiescent state by alveolar epithelial cells through mutual interactions involving surface proteins and mediators release (including the antiinflammatory cytokines IL-10 and TGF-β). Upon pathogens sensing, this regulatory interaction is lost, and an inflammatory response is initiated via AM activation (Bissonnette et al., 2020). We cannot exclude the same process to take place during crystalline silica exposure. Besides AM, inhaled crystalline silica also affects other antigen presenting cells (APC), including dendritic cells (DC). The activation of DC upon crystalline silica exposure induced the release of IL-6, IL-12 and TNF-α in murine models, suggesting that DC could influence innate or humoral immunity in the airways by modulating the release of proinflammatory molecules (Beamer and Holian 2007).

**FIGURE 1:**
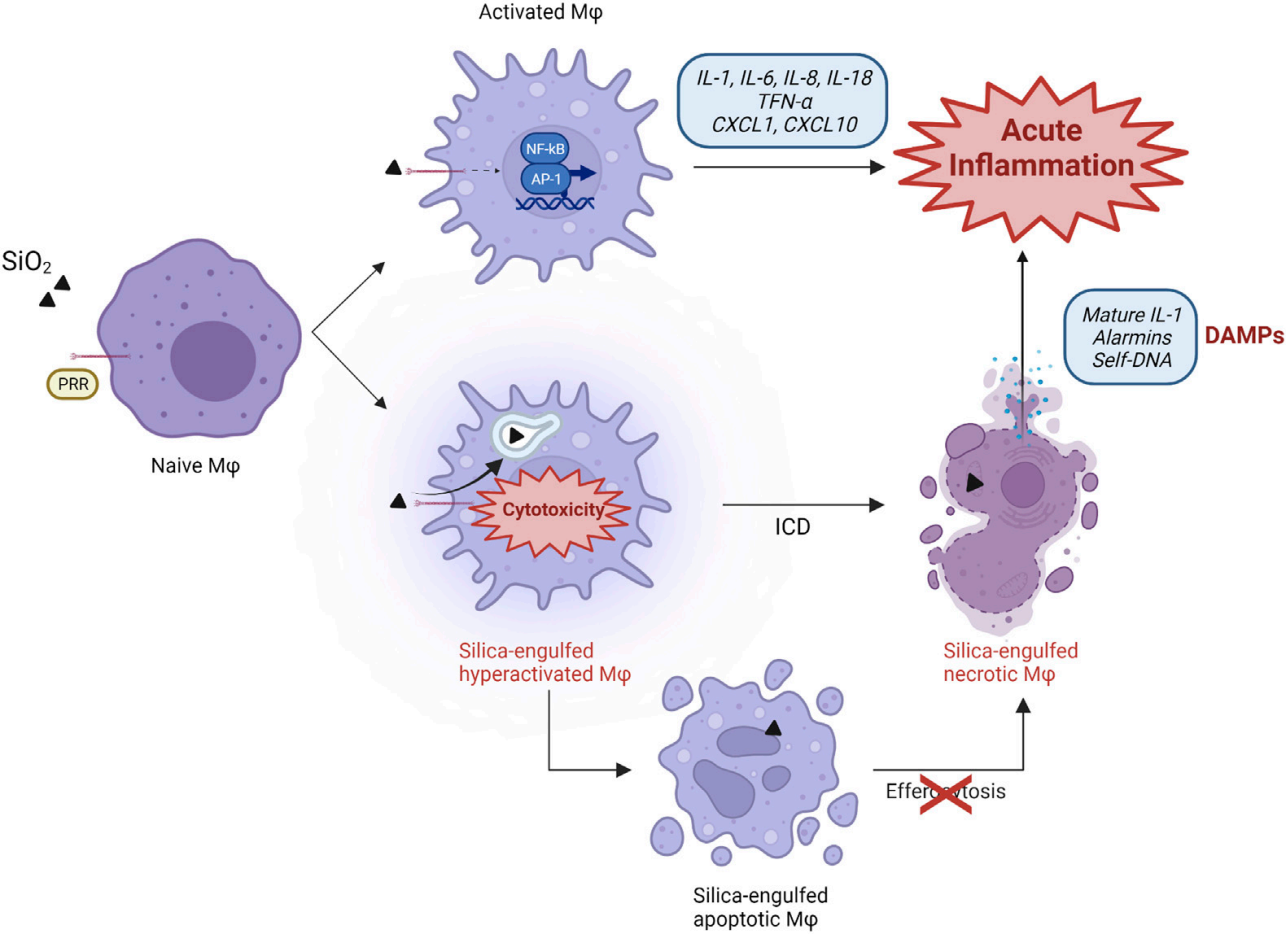
Proposed scenario in silica-induced lung acute inflammation. The classical mechanism explaining particle-induced inflammation is based on the recognition of silica particles by PRR receptors (SR) expressed on the surface of naïve alveolar macrophages. This sensing activates the transcription factors NF-kB and AP-1, which induce the expression, production, and release of several proinflammatory cytokines and chemokines (IL-1, IL-6, IL-8, IL-18, TNF-α, CXCL1, CXCL10). These molecules orchestrate a proinflammatory environment, characterized by the influx of additional immune cells. Immunogenic cell death (ICD) also participates in the development of inflammation, and represents an additional pathway characterized by silica engulfment, ROS generation, lysosome destabilization, and macrophage hyper-activation. This process results in the continuous release of self-DAMPs (mature IL-1, alarmins, DNA) and contributes to the inflammatory response to silica. Besides necrotic ICD, silica also induces apoptosis. This cell death modality progresses to proinflammatory secondary necrosis if apoptotic macrophages are not efficiently removed by phagocytic cells during efferocytosis, which can be impaired by silica particles.

Therefore, following the model currently accepted, the pathogenic effect of silica is mainly related to the activation of alveolar cells (macrophages and epithelial cells) resulting in cytokine, chemokine and oxidant production and the orchestration of a proinflammatory microenvironment (Figure 1). This process is mainly characterized by accumulation and activation of polymorphonuclear (PMN) leukocytes via inflammatory mediator release. The prolonged presence of the particles due to their biodurability elicits a long-term inflammatory detrimental cycle. In this context, investigations of crystalline silica dissolution kinetics showed that its dissolution rates are 10 times slower than amorphous SiO_2_ particles (Crundwell 2017; Benmerzoug et al., 2019). The mechanism driving silica dissolution is still unknown, but it could explain the long retention time of crystalline particles in the lungs, leading to chronic inflammation (Kawasaki 2015). Chronic inflammation is detrimental for the tissue because of the excessive production of cytotoxic granule proteins by neutrophils and eosinophils, possibly contributing to the extension of silica-induced damage (Gigon et al., 2021).

## Cytotoxic Particles and the New Immunogenic Cell Death Concept

Several studies demonstrated that, because of their intrinsic physico-chemical characteristics, reactive amorphous and crystalline silica particles are strongly cytotoxic and destabilize cell membranes inducing membranolysis (Ghiazza et al., 2010; Kawasaki 2015; Pavan and Fubini 2017). The early intracellular events are well defined and described from *in vitro* and *in vivo* investigations, and include production of oxidants, mitochondrial depolarization, lysosomal damage and inflammasome activation (Pavan and Fubini 2017; Borm et al., 2018; Wu et al., 2020). Nevertheless, the exact steps connecting cytotoxicity-induced cell death with the pathophysiological consequences of this process are far less understood in particle toxicology. Recent reports newly support the concept that silica induces persistent immunogenic cell death (ICD). ICD, therefore, is suggested to be implicated in the pathogenic activity of silica by modulating the innate and adaptive immune responses to particles via the release of DAMPs, and innate receptors activation (Figure 1). This repeated cycle is, in our view, pivotal to drive the development of SiO_2_-induced inflammatory and autoimmune diseases, and cancer. To this purpose, we first summarize the hallmarks of the different ICD modalities that have been so far observed after silica exposure. Then, we discuss the most recent and significant findings concerning *in vitro* and *in vivo* cell death modalities induced by silica in connection with ICD, DAMP and sensing concepts.

### Pyroptosis: From Inflammasome Activation to Alarmin Release

The term pyroptosis was introduced by Cookson and Brennan more than 20 years ago (Cookson and Brennan 2001). Pyroptosis is a caspase-regulated cell death form, which usually occurs in immune cells upon infection (Doitsh et al., 2014; Wang et al., 2019). It is considered as the prototype of inflammatory cell death since the inflammasome machinery guides the related molecular mechanism. The inflammasomes are key multiprotein complexes composed of initiating sensors (NLRP1, NLRP3, NLRC4, AIM2, or pyrin) characterized by the presence of the inflammasome adaptor protein ASC (Man et al., 2017). Inflammasomes activation takes place by canonical or non-canonical pathways, and mediates caspase-1 (casp-1) activation, a cysteine protease promoting maturation and secretion of proinflammatory IL-1β and IL-18 (Franchi et al., 2009). Casp-1 is the major regulator of pyroptosis, which can be modulated by other caspases such as casp-11 (casp-4 in human) (Kayagaki et al., 2011). It was recently discovered that casp-1 activation triggers the formation of pores in the plasma membrane, in particular via the family member gasdermin-D (GSDMD). These pores allow the release of intracellular components, dissipate the cytosolic ionic gradient (Fink and Cookson 2006; Liu et al., 2020) and permit the secretion of proinflammatory cytokines and alarmins from dying macrophages (Mossman and Churg 1998; Peeters et al., 2013; Rabolli et al., 2016; Evavold et al., 2018). Interestingly, significant reduction in TNF-α and IL-6 secretion was detected in casp-1 deficient macrophages in response to LPS stimulation, suggesting that casp-1 activation regulates the release of different cytokines besides IL-1β and IL-18 (Kuida et al., 1995; Li et al., 1995). This effect also includes the alarmins IL-1α and HMGB1 (Phulphagar et al., 2021; Tsuchiya et al., 2021). It is recognized that pyroptotic-related NLRP3 inflammasome activation is additionally stimulated by the release of lysosomal enzymatic content, particularly cathepsin B, following lysosomal membrane permeabilization (LMP). This process was recently recognized to be a key inducer of several cell death modalities (Wang et al., 2018; Alu et al., 2020).

All the processes related to pyroptosis are commonly observed *in vitro* during the exposure of phagocytes to cytotoxic silica. Upon LMP, NLRP3 inflammasome and casp-1 activation, immunogenic pyroptosis associated with the release of IL-1β was induced in alveolar macrophages after crystalline particle phagocytosis (Cassel et al., 2008; Hornung et al., 2008; Jessop et al., 2017; Nakayama 2018; Barnes et al., 2019) (Figure 1). Consistently, silica-exposed macrophages deficient in NLRP3 inflammasome components were unable to release IL-1β and IL-18 (Cassel et al., 2008). These successive events, accompanied by the persistence of inhaled particles in the alveolar microenvironment, result in the sustained release of other proinflammatory mediators including alarmins and nuclear proteins (IL-1α, HMGB1) (Brown et al., 2005; Kusaka et al., 2014; Pollard 2016; Mayeux et al., 2018; Zhang et al., 2018; Du et al., 2019). Recently, GSDMD-driven pyroptosis following highly pure crystalline silica exposure was detected in murine peritoneal macrophages in association with the release of IL-1β and LDH as biomarker of membranolysis (Song et al., 2022). Comparable outcomes were observed in other cells including primary airway epithelial cells (AEC) exposed to microcrystalline silica (Chen et al., 2021). Pyroptosis is additionally induced by amorphous silica via similar mechanisms comprising LMP and casp-1 activation. However, the pyroptotic responses triggered by amorphous particles larger than 1000 nm seems to be less robust in comparison to what observed with smaller particles (Kusaka et al., 2014). Comparable outcomes, including ROS production, GSDMD cleavage and IL-1β release, were observed with amorphous Stöber nanosilica in murine primary microglia cells and J774 macrophages. These observations support the hypothesis that both surface reactivity and particle size play fundamental roles in the activation of the inflammasome machinery and subsequent pyroptotic ICD.

### Necroptosis and Other Necrotic Immunogenic Cell Death Modalities

Besides pyroptosis, other modalities of silica-induced proinflammatory necrosis have been detected, including necroptosis and, in few studies, NETosis and ferroptosis.

Necroptosis is a form of necrosis triggered by several stimuli including activation of death receptors (Fas and TNFRSF1A), toll-like receptors (TLR3 and TLR4), and intracellular nucleic acid sensors (ZBP1, STING). It was proposed as a cell death modality able to remove pathogen-infected cells resistant to apoptosis (Choi et al., 2019). Therefore, it is accepted that necroptosis triggers immune system activation for boosting damaged cell clearance and inflammatory pathologies such as amyotrophic lateral sclerosis, multiple sclerosis, and Crohn disease (Conrad et al., 2016; Dhuriya and Sharma 2018). A potent molecular regulator of necroptosis is casp-8, an initiator caspase also implicated in extrinsic apoptosis (Fritsch et al., 2019). In this context, necroptosis is hypothesized to act as a secondary cell death pathway when apoptosis is prevented (i.e. in virus infected cells, or cells treated with apoptosis inhibitors) (Dhuriya and Sharma 2018; Paludan et al., 2019). Necroptosis is initiated upon the activation of RIPK3, which in turns phosphorylates the pseudokinase MLKL. The activate form of MLKL compromises the integrity of the plasma membrane by interacting with phosphatidylinositol phosphates and, as a consequence, it induces plasma membrane permeabilization and eventually complete cell lysis (Dondelinger et al., 2014; Wang et al., 2014). Several proinflammatory DAMPs are released from necroptotic cells, including alarmins (HMGB1, IL-1α and IL-33), ATP, mitochondrial DNA, cytokines and chemokines (IL-6, CXCL1, CXCL2, CCL2) (Choi et al., 2019; Orozco et al., 2019). Necroptosis is classified into three different categories, namely extrinsic (stimulated by TNF-α), intrinsic (stimulated by reactive oxygen species; ROS), and intrinsic ischemia-mediated (stimulated by death-receptors activation) (Dhuriya and Sharma 2018). The first subtype represents the classical necroptosis where the necrosome, a multiprotein complex, is assembled after RIPK1 and RIPK3 interaction (Chen J. et al., 2019). Other proteins such as TLR3/4, TRIF and DAI also interact with RIPK3 to form the non-classical necrosome, triggering in this way the non-classical necroptosis. TNF, FasL, TRAIL and TWEAK have been classified as initiators of necroptosis (Chen J. et al., 2019). Also, interferons (IFNs) induce MLKL phosphorylation and trigger necroptosis upon detection of viral/bacterial components via intracellular DNA sensor activation (Brault et al., 2018; Chen et al., 2018). PAMPs like LPS have been shown to trigger necroptosis, by activating TLR4 signaling. Necroptosis progression is triggered by lysosomes through the release of cathepsins D and B following LMP induced by ROS or Ca^2+^-dependent enzymes (Alu et al., 2020).

The typical features of this alternative modality of inflammatory RCD modality were observed *in vitro* after crystalline silica exposure in human neutrophils and kidney cells (HK2) (Desai et al., 2017; Honarpisheh et al., 2017), and upon amorphous silica exposure in hepatocellular carcinoma (HCC) cells. Desai and coworkers intriguingly observed that neutrophil necroptosis directly induced NETotic cell death (NETosis), a specific form of inflammatory lytic necrosis following the extracellular deposition of nuclear and mitochondrial DNA by neutrophils (NETs) as a response to pathogens through GSDMD-induced pores (Desai et al., 2017).

In a single study, ferroptosis was shown to be induced by silica. Ferroptosis is an additional immunogenic necrosis dependent on intracellular iron and lipid peroxide accumulation induced by ROS production and enzymatic mechanisms driven by lipoxygenases. Its potential proinflammatory activity depends on the release of classical inflammatory cytokines (Sun et al., 2020; Yu et al., 2021). Ferroptosis was induced by surface-functionalized ultrasmall silica nanoparticles (<10 nm in diameter) in cancer cells and cancer-bearing mice, resulting in the suppression of tumor growth (Kim et al., 2016). However, it is unknown if unmodified nanosilica particles with comparable size induce similar effects.

### DNA Sensors and DNA Sensing-Induced Cell Death

High levels of DNA fragments in the cytoplasm constitute a major danger signal for cells (Li and Chen, 2018). In homeostatic conditions, mislocalized DNA is digested by DNases located in the extracellular environment (DNase I), in endosomes (DNase II) and in the cytoplasm (DNase III). However, during viral and/or bacterial infections or following nuclear or mitochondrial damage, the large amount of free DNA accumulated in the cytoplasm is sensed by specific sensors, leading to a signaling cascade activating a broad range of stress responses resulting in cytokine release and cell death (Paludan et al., 2019).

In particular, the sensing of mislocalized self-DNA by Toll-like receptors 9 (TLR9) in endolysosomes or by the intracytoplasmic sensors cyclic GMP-AMP synthase (cGAS) and interferon-induced protein AIM2, governs the production of key inflammatory cytokines comprising IFN-I, IFN-III and IL-1β (Briard et al., 2020). TLR9 was the first observed PRR to bind preferentially unmethylated CpG DNA, which abundantly occurs in viruses and bacteria. Its activation results in the production of proinflammatory cytokines and IFN-α via NF-kB and IRF7 pathways (Hemmi et al., 2000). Similar outcomes were observed upon cGAS-STING activation by cytosolic double-strand viral DNA. cGAS, the nucleotidyl enzyme transferase, is the essential sensor, and its length-dependent interaction with double-strand DNA promotes the catalyzation of ATP and GTP into 2′3′-cGAMP (Cai et al., 2014). This second messenger is then able to bind and activate STING via conformational changes. STING is an endoplasmic reticulum membrane protein which, after being activated by cGAMP, translocates from the ER to the Golgi where it interacts with TBK1 or IKK and, in turn, activates a cascade of events resulting in the transcriptional expression of IFN-I and other inflammatory cytokines and chemokines including TNF-α and CXCL10 (Brault et al., 2018; Hopfner and Hornung 2020). Cytoplasmic double-strand DNA can also bind AIM2. Similarly to the NLRP3 inflammasome, AIM2 activation induces maturation and release of IL-1β and IL-18 from myeloid cells and, eventually, leads to pyroptosis via GSDMD-triggered membranolysis (Briard et al., 2020). Pyroptosis, like other RCD modalities including apoptosis and necroptosis, also results from cGAS-STING pathway activation. Indeed, cGAS trafficking to the lysosomes disrupts the lysosome membrane inducing K^+^ efflux and NLRP3 inflammasome activation (Gaidt et al., 2017). As a typical proinflammatory ICD modality, pyroptosis was also shown to cause self-DNA release after cell membrane disruption (Fink and Cookson 2006), which can be considered as a source of extracellular self-DNA *in vivo* (Benmerzoug et al., 2018).

Because cGAS-STING cascade is involved in several models of inflammatory lung diseases induced by cigarette smoke and allergens (Nascimento et al., 2019; Han et al., 2020), the possibility that silica induced a self-DNA-driven inflammatory responses was considered. In this context, Benmerzoug and coworkers demonstrated the crucial role of STING signaling in the activation of silica-exposed macrophages (Benmerzoug et al., 2018). In particular, they observed that *in vitro* release of IFN-I and CXCL10 triggered by DQ12 crystalline silica, was inhibited in STING^-/-^ BMDM. Interestingly, wild type BMDM activation was strongly reduced after silica exposure by degrading extracellular DNA with DNase I. These important observations strongly suggest that mislocalized self-DNA represents one of the key DAMPs related to silica toxicity (Figure 1). This hypothesis was elegantly investigated and supported by transfecting *in vitro* macrophages with self-DNA fragments obtained from the lungs of crystalline silica-exposed mice. Indeed, TNF release and IFN-I signatures were observed in DNA-transfected wild-type BMDM, but not in transfected STING^-/-^ cells. Unlike BMDM, DC responses were not affected by extracellular self-DNA degradation, suggesting DC activation depends on mitochondrial DNA (Benmerzoug et al., 2018). These results reveal the crucial function of self-DNA sensing in orchestrating the early immune responses to silica and suggest that the mechanisms of DNA-sensing may strongly vary among different cell types.

### Implication of Apoptosis and Secondary Necrosis as Immunogenic Cell Death

Besides necrosis, numerous studies report that silica induced apoptosis in *in vitro* and *in vivo* models. Apoptosis, the most well described RCD modality, takes commonly place in the absence of inflammation since apoptotic cells actively communicate with the surrounding environment, leading to antiinflammatory pathways activation (Martin et al., 2012). Nevertheless, it should not be considered as a completely silent cell death modality. Recent studies have shown that immunogenic apoptosis induced in tumor cells by chemotherapeutic agents or via physical methods stimulated some components of the immune system and enhanced immunological responses (Fogarty and Bergmann, 2015; Montico et al., 2018). In this context, experimental data suggest that an immunogenic form of apoptosis is triggered by certain anticancer therapies and can be beneficial for antitumor immunity (Pfeffer and Singh, 2018).

Extrinsic and intrinsic apoptotic pathways are so far described. The first subtype depends on membrane receptors signaling (Fas, TNFR1) and is initiated by the activation of casp-8 and casp-10 (initiator caspases). Furthermore, extrinsic apoptosis is induced by dependence receptors (UNC5B and DCC) through the activation of casp-9, or *via* DAPK1 dephosphorylation, a positive mediator of IFN-γ-mediated apoptosis (Singh et al., 2016). Intrinsic apoptosis occurs after mitochondrial outer membrane permeabilization in response to different stimuli. This process triggers the release of mitochondrial proteins in the cytoplasm (CYCS, DIABLO, HTRA2) and casp-9 activation. The final steps in apoptosis are driven by casp-3, casp-6 and casp-7, known as effector caspases. These enzymes cleave several substrates such as intracellular structural proteins, leading to the acquisition of a full apoptotic morphology. In addition, apoptosis was observed in response to intracellular adverse events such as LMP and cathepsin release in the cytoplasm, as a result of mitochondrial damage (Johansson et al., 2010; Oberle et al., 2010).

Amorphous and crystalline silica can both induce apoptotic cell death *in vitro* (Wilhelmi et al., 2012). In particular, amorphous silica nanoparticles increased pro-apoptotic members BAD and BAX, and decreased antiapoptotic protein BCL-2 in different cell types (Yang Y. et al., 2017; Lu et al., 2017; Guo et al., 2018; Liu and Tang, 2020). In amorphous silica-treated RAW 264.7 macrophages, Chen and others observed endoplasmic reticulum stress-related apoptosis and upregulation of pro-apoptotic proteins DDIT3 and casp-12 (Chen F. et al., 2019).

Apoptosis hallmarks including p53 and PAI-1 expression, and casp-3 activation, were observed in alveolar epithelial cells (AEC) isolated from crystalline silica exposed wild-type mice (Bhandary et al., 2015). *In-vitro* investigations with different cell types also supported crystalline silica-induced apoptosis driven by casp-3 and casp-9 (Wilhelmi et al., 2012; Wang et al., 2016; Hu et al., 2017; Rajasinghe et al., 2020). Intriguingly, it was also shown that Min-U-Sil 5 quartz induced signs of apoptosis and necrosis (LMP, caspase activation and cell blebbing) at the same time in MH-S alveolar macrophages (Joshi and Knecht 2013). Further observations suggested that crystalline silica-induced apoptosis results in phagosomal and mitochondrial damage likely regulated by intracellular ROS generation and p53 (Santarelli et al., 2004; Gwinn et al., 2009; Joshi et al., 2015). In some conditions, the apoptotic process following crystalline silica exposure was accompanied by endoplasmic reticulum stress (Hu et al., 2017) and significant expression of proinflammatory TNF-α *in vivo* (Pfeffer 2003; Attik et al., 2008).

Overall, these data indicate that amorphous and crystalline silica triggers different intracellular pathways, which can eventually lead to cell death via apoptosis. However, a direct confirmation that immunogenic apoptosis takes place as the result of silica exposure still lacks. Nevertheless, it is well recognized that apoptotic cells when not efficiently cleared out by phagocytes progress to secondary necrosis, resulting in loss of plasma membrane and immunostimulatory DAMPs release (Westman et al., 2019) (Figure 1).

### PANoptosis: An Emerging Concept Englobing Different Simultaneous Immunogenic Cell Death Modalities

The simultaneous activation of different cell death modalities and immunogenic regulated pathways was taken up through a new notion named PANoptosis. This process includes key features of pyroptosis, necroptosis and apoptosis, via the activation of the PANoptosome, a multimeric cytoplasmic complex consisting of several proteins involved in several ICD modalities (including casp-1, RIPK-1 and casp-3/-8) (Samir et al., 2020). Inflammatory PANoptosis was firstly observed in influenza A virus (IAV) infected cells (Kuriakose et al., 2016), where the immune sensing of viral nucleic acids by ZBP1 induced the activation of the PANoptosome machinery. In this context, the final executioners of pyroptosis, necroptosis and apoptosis (GSDMD, MLKL, and casp-3/-7, respectively), were activated in response to the PANoptosome formation, allowing PANoptosis to take place. PANoptosis was recently detected to occur during murine hepatitis virus (HMV) and vesicular stomatitis virus (VSV) infections (Christgen et al., 2020; Zheng et al., 2020), suggesting that the investigation of PANoptosis mechanism could open new horizons in understanding the pathways underlying the development of many viral diseases. In silica-exposed cells, the presence of PANoptosome has been hypothesized, but not yet fully addressed. Since many components of the PANoptosome have been associated to inflammation, autoimmune diseases and cancer, understanding whether silica induces PANoptosis would provide researchers the opportunity to better delineate silica pathogenicity.

## Implication of Immunogenic Cell Death in Silica-Related Diseases

The observations reported in the previous chapter suggest that *in vitro* cytotoxic activity of silica results in of the activation of several intracellular processes, which finally lead to ICD and proinflammatory DAMPs release. To elucidate the *in vivo* pathogenic potential of silica, it is crucial to clarify the relevance and significance of the *in vitro* available data. In this chapter, we debate the relationship between ICD, immune system modulation and the onset of crystalline SiO_2_ exposure-related diseases, proposing new insights on the mechanisms driving the development of detrimental human pathologies.

### Immunogenic Cell Death Drives Particle-Induced Neutrophilic Lung Inflammation and Fibrosis

As previously discussed, cells undergoing ICD emit a panel of immunostimulatory DAMPs and proinflammatory cytokines including alarmins, IL-1 related cytokines and IFN-I, accounting for a strong inflammatory response (Duan et al., 2019; Galluzzi et al., 2020; Chauhan et al., 2021).

**FIGURE 2:**
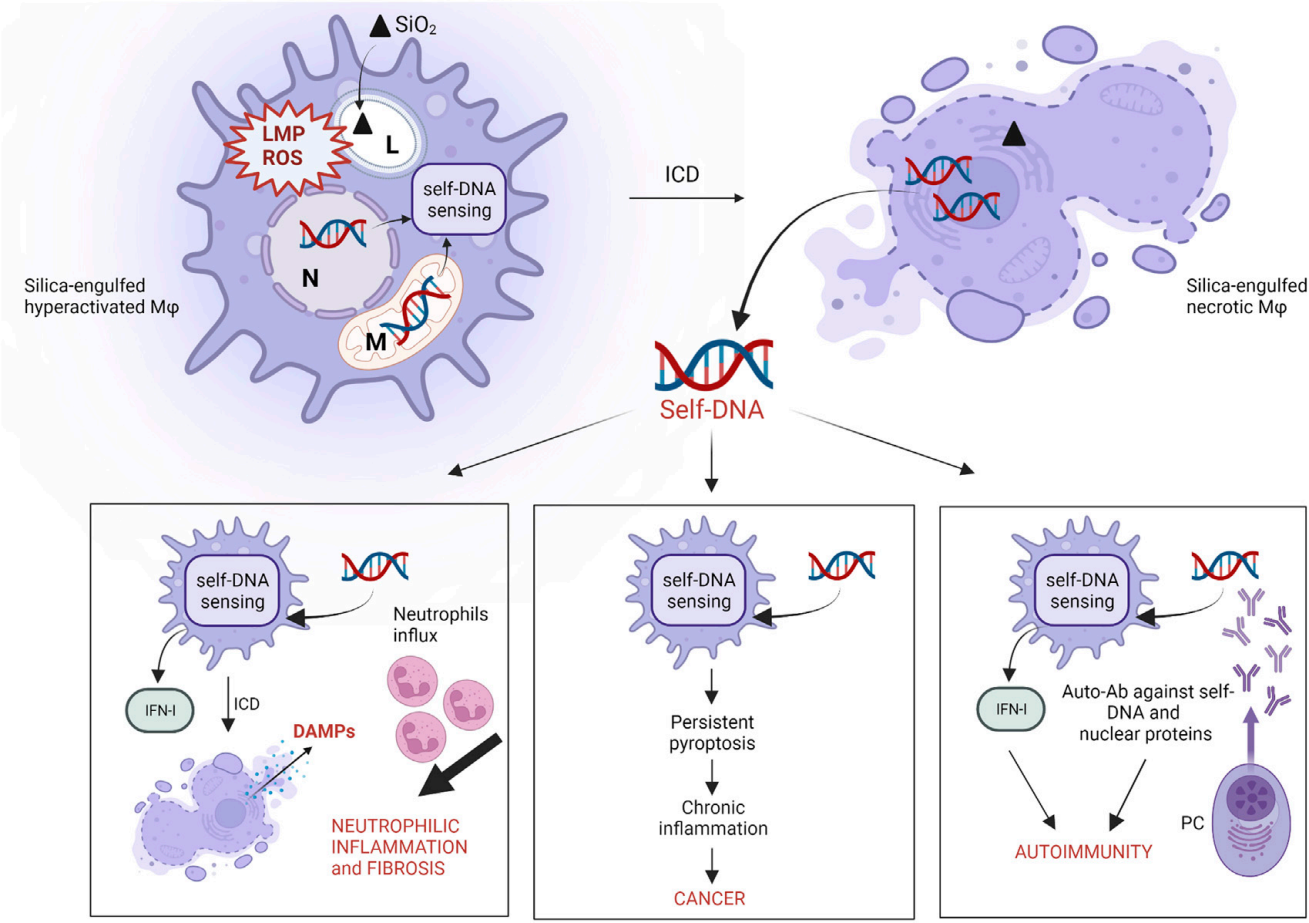
Proposed role of self-DNA sensing in silica-induced pathogenicity. The engulfment of silica particles by alveolar macrophages elicits cell stress as a result of lysosome membrane permeabilization (LMP) and sustained intracellular ROS production. These effects trigger mitochondrial membrane damage and/or micronuclei formation and rupture, due to incomplete chromatin segregation, which causes cytoplasmic accumulation of self-DNA fragments and DNA sensors (AIM2, cGAS-STING) activation. Particle cytotoxicity and DNA-sensing result in necrotic cell death (ICD), associated with self-DNA release in the extracellular environment. The internalization of these extracellular self-DNA fragments by “silica-free” macrophages induces DNA-sensing activation leading to neutrophilic inflammation and fibrosis in response to IFN-I, ICD and DAMPs release. Sustained self-DNA sensing due to cytotoxicity of biodurable silica particles induces persistent pyroptotic ICD, chronic inflammation and cancer. Extracellular self-DNA and nuclear proteins resulting from plasma membrane complete lysis following ICD trigger and/or exacerbate autoimmunity by inducing the secretion of anti-dsDNA and anti-nuclear protein autoantibodies by plasma cells (PC). (L: lysosome; N: nucleus; M: mitochondria).

Several *in vivo* studies support the reasonable hypothesis that necrotic ICD induced by crystalline silica drives and amplifies innate immune responses to silica. Indeed, ICD assessed by the release of LDH as biomarker of cell membrane lysis (Chan et al., 2013) is associated with the robust release of proinflammatory cytokines (IL-1β) and alarmins (IL-1, IL-33, IL-18, HMGB1) during neutrophilic inflammation and fibrosis (Barbarin et al., 2005; Rabolli et al., 2014; Lo Re et al., 2014; Pavan et al., 2020) (Figure 2). Recently, it was observed that GSDMD^-/-^ mice, deficient for pyroptosis, had down-regulated levels of proinflammatory cytokines IL-1β/IL-6 and less lung fibrosis, supporting the crucial role of GSDMD and pyroptotic ICD in pulmonary inflammation (Song et al., 2022). These recent data support what previously observed by Cassel and coworkers using crystalline silica-treated ASC-/NALP3-/IL1β deficient mice, which showed less lung inflammation and fibrosis (Cassel et al., 2008). Comparable effects were noticed by treating mice with an IL-1 receptor antagonist or IL-1β-neutralizing monoclonal antibodies after exposure to c-SiO_2_. (Piguet et al., 1993; Guo et al., 2013). Furthermore, the genetic depletion of IL-1α inhibited mature- and pro-IL-1β expression, and strongly reduced lung neutrophilic inflammation in response to DQ12 quartz (Rabolli et al., 2014). These results clearly demonstrate the crucial role of necrotic ICD and pyroptotic machinery in orchestrating the *in vivo* inflammatory and pathogenic responses to crystalline silica.

Additional pathways related to cell death and ICD also explain silica-induced lung inflammation. Mice exposed to c-SiO_2_ exhibited IFN-I inflammation coordinated by self-DNA sensing (Benmerzoug et al., 2018; Benmerzoug et al., 2019) (Figure 2). Indeed, IFN-I and IRF-7 signatures were detected during the long-term responses to silica in mice. Interestingly, long-term chemokine production and neutrophilic inflammation were dramatically reduced in IFNAR- and IFR-7-deficient mice. In this model, pulmonary fibrosis onset was not affected, suggesting that under certain conditions, fibrosis can occur regardless of inflammation (Giordano et al., 2010). Crystalline silica-activated IFN-I response is dependent on cGAS-STING activation by self-DNA (Benmerzoug et al., 2018; Kato et al., 2018; Benmerzoug et al., 2019). Treatment of mice with DNase I reduced self-DNA levels, inhibited STING activation and suppressed the associated IFN-I signature, suggesting the central role of self-DNA in crystalline silica-induced lung inflammation, through the activation of cGAS-STING pathway (Benmerzoug et al., 2018). Also, it was demonstrated that silica-induced lung inflammation associated to IFN-I was drastically reduced in cGAS-STING deficient mice (Benmerzoug et al., 2018; Benmerzoug et al., 2019). Patients suffering from silicosis showed increased plasma levels of dsDNA and higher concentrations of CXCL10 in their sputum, indicating that the same mechanism observed in mice is probably active in humans (Benmerzoug et al., 2018).

In addition to necrotic ICD, crystalline silica exposure induces apoptosis during lung inflammation in rodents (Leigh et al., 1997; Srivastava et al., 2002). This might be surprising, because it is known that apoptotic cells are generally associated to non-inflammatory conditions *in vivo*. Cell debris produced by apoptosis are recognized and removed by resident macrophages through a homeostatic process called efferocytosis, which exerts strong immunosuppressive effects (Elliott et al., 2017). Efficient efferocytosis is essential for tissue maintenance and is crucial in the resolution of inflammation. On the contrary, defective or impaired efferocytosis and accumulation of apoptotic cells result in ICD through secondary necrosis, which is characterized by the release of proinflammatory immunogenic DAMPs (proteases, nucleosomes and alarmins) after membrane rupture of apoptotic bodies (Sachet et al., 2017). Extensive secondary necrosis due to impaired efferocytosis results in long-term tissue accumulation of intracellular agents and cell debris. Sign of defective efferocytosis and consequent secondary necrosis have been observed in several inflammatory pathologies such as systemic chronic obstructive pulmonary disease (COPD), autoimmune disorders including systemic lupus erythematosus (SLE) and atherosclerosis (Savill et al., 2002; Munoz et al., 2010; Silva 2010; Abdolmaleki et al., 2018; Kawano and Nagata 2018). However, the precise role of secondary necrosis in the onset of these diseases still needs to be examined in detail. It was recently observed that crystalline silica strongly inhibits the efferocytosis ability of human monocyte-derived macrophages by a mechanism depending on SR-B1 membrane receptor expression (Lescoat et al., 2020). Similarly, the *in vitro* phagocytic activity of cultured murine macrophages (RAW264.7) was affected after repeated exposures to amorphous silica particles, suggesting that both types of silica induce a comparable effect in modulating the efferocytosis activity of phagocytes (Torres et al., 2020).

The dampening of efferocytosis explains *in vivo* inflammation following secondary necrosis in the event of unresolved apoptosis, a process occurring when the ingestion of apoptotic cells and debris by phagocytes is delayed or prevented. Efferocytosis inhibition justifies the simultaneous presence of apoptotic and necrotic cells in silica-exposed animal models. However, since DAMPs released after secondary necrosis differ from those released during primary necrosis, the exact inflammatory capacity of secondary necrosis-associated DAMPs should be deeply investigated. (Sachet et al., 2017).

### Immunogenic Cell Death and Silica-Related Autoimmune Diseases (AID)

Several epidemiological studies have reported significant correlation between occupational exposure to crystalline silica and autoimmune disorders such as rheumatoid arthritis (RA), systemic lupus erythematosus (SLE) and systemic scleroderma (SSc) (Shtraichman et al., 2015; Chen et al., 2016; Pollard 2016). To date, no complete clarification has been established on the mechanism explaining how crystalline silica interacts with the immune system and induces AID. However, it was recently proposed that the onset/exacerbation of autoimmune diseases by crystalline silica could rely on ICD and its consequences (Pollard 2016) (Figure 2). As mentioned in the above section, silica-induced necrosis is associated with IFN-I production through the activation of DNA sensors and induces acute neutrophilic inflammation *in vivo*. In this context, elevated self-DNA levels were observed in the serum of SLE and SSc patients compared to healthy controls, and impaired self-DNA degradation was associated with IFN-I-driven chronic inflammation (Kato et al., 2018; Vlachogiannis et al., 2020). The scenario where crystalline silica initiates and amplifies autoimmunity via extracellular self-DNA accumulation after ICD should be therefore considered.

These findings are consistent with silica-associated autoimmune responses because IFN-α and -β are known to orchestrate the successive events leading to AID i.e. proinflammatory cytokine production, innate and adaptive immunity activation, tolerance breaking, autoantibodie production and tissue damage (Lauwerys et al., 2014; Pollard 2016). The concept linking cell components released after ICD and AID development seems attractive, as autoantibodies directed against self-nuclear and cytoplasmic proteins serve as clinical diagnostic and prognostic markers of several AID (Castro and Gourley 2010). Higher frequency of autoantibodies (anti-DNA, anti-SS-A/Ro, anti-SS-B/La, anticentromere and anti-DNA topoisomerase 1) has been found in silica-exposed patients in comparison with the general population (Denton and Khanna 2017; Hoy and Chambers 2020). Furthermore, autoimmune manifestations, including anti-nuclear and anti-dsDNA autoantibodies were detected in silica-exposed mice in accordance with increased levels of lung LDH, suggesting a clear association between silica-induced ICD and autoimmune responses (Mayeux et al., 2018).

### SiO_2_-Induced Immunogenic Cell Death in Cancer Development

In 1987, the International Agency for Research on Cancer (IARC) classified crystalline silica as a probable carcinogen. Later, in 1997, crystalline silica was re-classified as a Group 1 carcinogen (IARC 1997). Several epidemiological studies demonstrate that silica exposure is associated with increased risk of lung cancer in humans (Poinen-Rughooputh et al., 2016; Pollard 2016). Carcinogenesis was shown to be a consequence of silica-induced prolonged inflammation (Sato et al., 2018). Sustained inflammation due to the continuous influx of oxidant-producing activated macrophages and neutrophils exacerbates lung epithelial cell damage upon the pulmonary accumulation of biopersistent silica particles (Sato et al., 2018). These processes lead to uncontrolled genotoxic damage, which may ultimately trigger tumor formation. Despite several mechanisms have been proposed, the exact pathway leading to the initiation/promotion of *in vivo* silica-related carcinogenesis is still unknown. However, according to literature data, silica-induced ICD and DNA sensing could play a significant role in this process (Figure 2). Indeed, significant correlation between the expression/secretion of identified ICD-associated DAMPs (including alarmins, such as HMGB1, and self-DNA) and the presence of tumors was outlined in *in vivo* models (Sims et al., 2010; Hernandez et al., 2016; Kwon and Bakhoum 2020). Early and sustained genotoxicity evidenced by the accumulation of specific DNA-damage biomarkers including γH2AX and pCHK2, was recently detected in lung homogenates of silica-treated mice (Wu et al., 2020). Interestingly, it was hypothesized in this study that silica-induced DNA damage is triggered by the AIM2-driven sensing of self-DNA fragments. This conclusion was supported by *in vitro* observations in silica-exposed 16HBE and A549 cells, suggesting a possible tumor-promoting effect of AIM2-guided sensing (Yu Q et al., 2019). In addition, biomarkers of pyroptotic ICD, including IL-1β release, were detected in the lungs of NNK (a potent carcinogen presents in cigarette smoke) and silica co-exposed mice (Bode et al., 2014). Positive association between IL-1β gene expression and lung tumor development was also found, suggesting that silica-induced ICD increases lung cancer susceptibility (Bode et al., 2014). In the same vein, the exacerbation of tumor incidence through an intricate interplay between pyroptosis-associated cytokines (IL-1β), chemotactic cytokines (CXC- and CC- family members) and other chemoattractants (LTB4) released by macrophages, epithelial cells and mast cells was observed in Kras murine models (Satpathy et al., 2015). These observations might suggest an existing relationship between the events associated to silica-induced ICD, including DNA-sensing and DAMPs release, with acute/chronic inflammatory responses and tumor progression.

Besides being related to inflammation-mediated tumorigenesis (Kwon and Bakhoum 2020), it is recognized that DNA sensing via cGAS-STING may result in anti-tumorigenic effects (Li and Chen 2018; Gong et al., 2020; Kwon and Bakhoum 2020). In this context, STING agonists were proven to promote tumor control and the efficacy of this anti-tumoral treatment was reduced in cGAS-STING deficient mice (Li and Chen 2018; Paludan et al., 2019). Similarly, DAMPs released upon ICD induced by specific anti-cancer agents foster a potent anti-tumor immune effect (Hernandez et al., 2016). In particular, the engagement of several DAMPs, including HMGB1, with receptors present on immune cells activates a strong anti-tumor immunity. Other studies additionally demonstrated that the reduction of DAMP release inhibited the observed anti-tumor response (Apetoh et al., 2007; Michaud et al., 2011). For these reasons, further studies are needed to detail the role played by DNA sensing and identify the set of DAMPs secreted in carcinogenic models upon silica exposure.

## A New ICD-Driven Scenario In Particle Toxicology: DNA Sensing As The Crucial Stage

Taking advantage of *in vitro* and *in vivo* data discussed in this review, we propose self-DNA and self-DNA sensing as crucial players in the onset of crystalline silica-induced diseases, including lung inflammation, cancer and AID (Figure 2). In this updated scenario, the massive pulmonary cell stress resulting from silica inhalation induces the intra-cytoplasmic accumulation of self-DNA fragments as a result of mitochondrial and/or nuclear damage (Harding et al., 2017; Mackenzie et al., 2017; Yu Y et al., 2019; Pal et al., 2020). The subsequent activation of self-DNA sensing leads to proinflammatory ICD and self-DNA fragment release in the extracellular milieu following plasma-membrane lysis. Mislocalized extracellular self-DNA fragments gain access to cytoplasmic sensors of “silica-free” surrounding cells supporting inflammation in the lungs via the activation of IFN-I responses that promote additional ICD and neutrophilic inflammation. The presence of extracellular self-DNA might also explain the secretion of anti-self-DNA autoantibodies by plasma cells, together with autoantibodies directed towards nuclear proteins, as observed in several studies investigating the autoimmune potential of silica. In addition, persistent IFN-I responses resulting from crystalline silica-induced DNA-sensing can support autoimmunity and cancer.

Although the consistency of literature data, ad-hoc studies are warranted to address the precise molecular connections between silica-induced self-DNA-dependent signaling, immune responses and inflammatory diseases onset. Furthermore, even though several mechanisms have been proposed, including exosomes, cargo chemokines and apoptotic bodies engulfment, the way extracellular self-DNA enters cells and becomes available for intracellular DNA sensors is still unclear and should be thoroughly investigated (Couillin and Riteau 2021).

## DAMPs, AOP and Predictive Tests

This review highlighted that the pathogenic activity of silica, especially in crystalline form, relies on the modulation of the immune system by a plethora of damage signals (DAMPs) released in the extracellular environment after silica-induced ICD (Figures 1, 2). In this context, the central role of DAMPs sensing following ICD may be proposed as a new and first molecular and cellular key event in the adverse outcome pathway (AOP) scheme for silica, enriching the model recently described by Pavan (Pavan and Fubini 2017).

The intrinsic variability of silica physico-chemical properties and the possibility to modulate these properties through functionalization strategies (Marzaioli et al., 2017; Pavan et al., 2020) deserve reliable and rapid screening tests. In compliance with the principles of the 3Rs, the *in vivo* pathogenic effect of pristine and modified silica particles was first evaluated *in vitro* through bioassays investigating the production and release of master cytokines and chemokines from particle-activated macrophages or other cells (e.g., TNF-β, IL-1β, IL-6, CCL2, IL-8). The rapid advances in ICD assessment and the discovery of silica cytotoxicity-associated DAMPs, including IL-1α (Rabolli et al., 2014) and self-DNA (Benmerzoug et al., 2018), provide predictive toxicology exciting and convenient new options to move towards a combination of screening tests. This approach aims the detection of diverse immune biomarkers resulting from cell activation or ICD. Besides silica, the global strategy here proposed can be extended to other fine and ultrafine particles, including microplastics and urban particles, with unknown or contradictory pathogenic potential. In this context, the combination of different tests ensures a complete safe-by design approach in the development of new nanomaterials for industrial or medical purposes, allowing a complete safety and biocompatibility screening.

## Glossary

16HBE: Human bronchial epithelial cells 16HBE14o
AID: Autoimmune disease
AIM2: Interferon-inducible protein AIM2
APC: Antigen presenting cells
ASC: Apoptosis-associated speck-like protein containing a CARD
BMDM: Bone marrow-derived macrophages
cGAS: Cyclic GMP-AMP synthase
c-SiO_2_: Crystalline silica
COPD: Chronic obstructive pulmonary disease
CXCL10: C-X-C motif chemokine ligand 10
CYCS: Cytochrome c
DAI: DNA-dependent activator of IFN-regulatory factor
DAMPs: Damage-associated molecular patterns
DAPK1: Death-associated protein kinase 1
DC: Dendritic cells
DCC: DCC Netrin 1 receptor
DDIT3: DNA damage-inducible transcript 3
DIABLO: Diablo IAP-binding mitochondrial protein
ERK1/2: Extracellular signal-regulated kinases 1/2
FasL: Fas ligand
GPCR: G-protein coupled receptors
GSDMD: Gasdermin-D
HTRA2: HtrA serine peptidase 2
γH2AX: γH2A histone family member X
ICD: Immunogenic cell death
IFN: Interferon
IFN-I: Type-I Interferon
IKK: IkappaB kinase
IRF7: Interferon regulatory factor 7
LMP: Lysosome membrane permeabilization
MAPK: Mitogen-activated protein kinase
MerTK: Proto-oncogene tyrosine-protein kinase MER
MLKL: Mixed lineage kinase domain-like
NF-kB: Nuclear factor-kappa B
NNK: 4-(methylnitrosamino)-1-(3-pyridyl)-1-butanone
PAMPs: Pathogen associated molecular patterns
PCHK2: Phosphorylated checkpoint kinase 2
PMN: Polymorphonuclear leukocytes
PRR: Pattern recognition receptors
RA: Rheumatoid arthritis
RAGE: Receptors for advanced glycation
RCD: Regulated cell death
RIPK3: Receptor-interacting serine/threonine kinase 3
ROS: Reactive oxygen species
SiO_2_: Silicon dioxide (silica)
SC: Scavenger receptors
SFK: Src family of protein tyrosine kinases
SLE: Systemic lupus erythematosus
SR-B1: Scavenger receptor class B type 1
SSc: Systemic sclerosis
STING: Stimulator of interferon genes
TBK1: TANK-binding kinase 1
TLR: Toll like receptor
TNF: Tumor necrosis factor
TNFR1: Tumor necrosis factor receptor 1
TNFRSF1A: TNF receptor superfamily member 1A
TRAIL: Tumor necrosis factor ligand superfamily member
TREM: Triggering receptors expressed on myeloid cells
TRIF: TIR-domain-containing adapter-inducing interferon-β
TSLP: Thymic stromal lymphopoietin
TWEAK: Tumor necrosis factor-related weak inducer of apoptosis
UNC5B: Unc-5 netrin receptor B
ZBP1: Z-DNA binding protein 1
